# Reconstruction of *Danio rerio* Metabolic Model Accounting for Subcellular Compartmentalisation

**DOI:** 10.1371/journal.pone.0049903

**Published:** 2012-11-14

**Authors:** Michaël Bekaert

**Affiliations:** Institute of Aquaculture, University of Stirling, Stirling, Scotland, United Kingdom; Medical College of Wisconsin, United States of America

## Abstract

Plant and microbial metabolic engineering is commonly used in the production of functional foods and quality trait improvement. Computational model-based approaches have been used in this important endeavour. However, to date, fish metabolic models have only been scarcely and partially developed, in marked contrast to their prominent success in metabolic engineering. In this study we present the reconstruction of fully compartmentalised models of the *Danio rerio* (zebrafish) on a global scale. This reconstruction involves extraction of known biochemical reactions in *D. rerio* for both primary and secondary metabolism and the implementation of methods for determining subcellular localisation and assignment of enzymes. The reconstructed model (ZebraGEM) is amenable for constraint-based modelling analysis, and accounts for 4,988 genes coding for 2,406 gene-associated reactions and only 418 non-gene-associated reactions. A set of computational validations (i.e., simulations of known metabolic functionalities and experimental data) strongly testifies to the predictive ability of the model. Overall, the reconstructed model is expected to lay down the foundations for computational-based rational design of fish metabolic engineering in aquaculture.

## Introduction

High blood levels of long-chain omega-3 fatty acids (n-3 FAs), eicosapentaenoic acid (EPA, 20∶5) and docosahexaenoic acid (DHA, 22∶6), decrease risk of human cardiovascular disease events [1]. Intake of fatty, cold-water fish such as salmon, mackerel, and sardines, or fish oil capsules are the most effective methods of increasing EPA and DHA levels in the blood [2]. Fish metabolic engineering predominantly involves fisheries, however the fish farming industry has been criticised for being a net consumer of marine resources, in the form of fishmeals and fish oils used in feeds, despite the efforts made to replace them with alternatives, such as vegetable proteins and oils [3].

Current challenges in using fish as factories for public health nutrition and nutraceuticals require predesigned and efficient strategies for metabolic engineering [4]. Currently, fish metabolic engineering mostly involves trial-and-error approaches, without the utilisation of computational modelling procedures to rationally design genetic modifications. The marginal contribution played by metabolic modelling in fish until now stands in marked contrast to its prominent success in plant and microbial metabolic engineering [4]–[6]. The reconstruction process is well established for metabolic networks [7]. Once assembled, the reconstruction can be readily converted into a mathematical format by adding balances (*e.g.*, mass-balance constraints), steady-state assumptions [8]. The resulting model is condition-specific and can be used for phenotype simulations using various constraint-based reconstruction and analysis methods [8], [9]. This approach has proven successful for numerous microorganisms [10] and eukaryotes for addressing various biological and biotechnological questions, such as the analysis of knowledge gaps [11], simulation of phenotype traits [12], analysis of evolution of metabolic networks [13], [14] and metabolic engineering applications [15]. These analyses solely rely on simple physical-chemical constraints, thus overcoming the problem of missing enzyme kinetic data [5]. Numerous applications for large-scale plant and microbial networks have proven to be highly successful in predicting metabolic phenotypes in metabolic engineering and many other applications [5], [16]. The reconstruction of metabolic network models for multicellular eukaryotes is significantly more challenging than that of bacteria, because of the larger size of the networks, the subcellular compartmentalisation of metabolic processes, and the considerable variation in tissue-specific metabolic activity [17].

A significant step forward in fish metabolic modelling was made with the publication of large-scale MetaFishNet model analysing high throughput expression data, as well as a framework for future systems studies [18]. This genome-wide model offered a significant expansion of the Kyoto Encyclopedia of Genes and Genomes (KEGG) zebrafish model [19]. In this work we present a computational reconstruction of genome-scale, subcellular compartmentalised metabolic network model for *D. rerio* (ZebraGEM), which relied on genomic and biochemical data extracted from various databases and literature. The validity of the model reconstruction steps is demonstrated via computational tests (cross-validation tests and simulations of known metabolic functionalities).

In this study, we present the reconstruction of the global *D. rerio* metabolic map. ZebraGEM is a comprehensive literature-based genome-scale metabolic reconstruction using manual curation, including extensive gap-assessment and filling. ZebraGEM has 9 compartments (cytoplasm, endoplasmic reticulum, extracellular space, Golgi apparatus, lysosome, mitochondria, mitochondrial intermembrane space, nucleus and peroxisome), 18 interfaces (surface orientations) and accounts for the functions of 4,988 proteins, 1,553 metabolites, and 2,824 metabolic and transport reactions. This network reconstruction was transformed into an *in silico* model and validated through the simulation of known metabolic functions found in a several tissue types.

## Results and Discussion

### Global Model Reconstruction

We adopted a reaction-centered view of *D. rerio* metabolism, where each metabolic gene is assigned to one or more reactions. We used Enzyme Commission numbers [20] to identify an initial set of metabolic genes from the KEGG database [19], National Center for Biotechnology Information’s Gene [21], and Reactome Database [22]. These genes were mapped to a rudimentary network of 1,374 metabolic enzymes and 1,550 reactions (see Methods). In addition we collected zerbafish-specific reactions from MetaFishNET [18]. The final *D. reria* metabolic model (ZebraGEM) was then reconstructed based on biochemical and physiological knowledge by surveying literature and mining databases. We evaluated more than 15 years of biological evidence from more than 1,800 primary literature articles, reviews, and biochemical textbooks. The reconstruction was almost entirely constructed from zebrafish-specific data and includes many reactions directly extracted from the literature that are not described in any chart or database. Furthermore, it represents carefully formulated metabolites and reactions, which account for known reaction stoichiometry, substrate/cofactor specificity, and directionality, as well as overall conservation of mass and metabolite ionisation states at pH 7.2. Unambiguous metabolite names were verified using Chemical Identifier Resolver [23].

### Compartmentalised Model Reconstruction

To extend the genome-scale *D. rerio* model reconstructed in the above step and to account for subcellular compartmentalisation of metabolic processes, we considered the known sub-localisation of the reactions taken from literature articles and reviews. Manual literature-based reconstruction ensured that the network components and their interactions were based on direct biochemical and subcellular evidence and reflected the current knowledge of *D. rerio* metabolism (see Methods).

**Figure 1:**
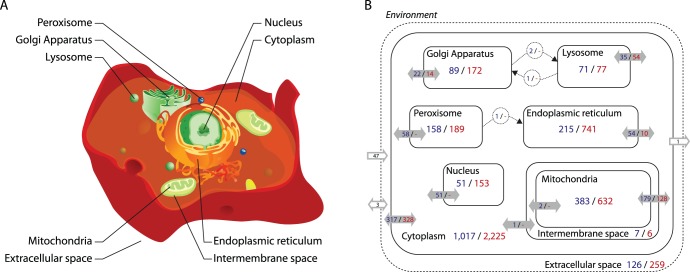
Overview of the metabolic network of *Danio rerio*, ZebraGEM. (**A**) Schematic of the 9 different compartments considered and their interfaces. (**B**) Break down of the reactions distribution (in blue) and associated genes (in red) for each compartment. Environmental exchanges are shown in grey-border arrows, biomass export as output, uptakes as input and oxygen, water and carbon dioxide freely exchange as doubled arrows. Solid-grey arrows denote reactions across membranes, with number of reactions and genes respectively indicated. Circles details vesicular transports.

The analysis resulted in a compartmentalised model (Figure 1), with each enzyme localised between eight intracellular spaces (cytoplasm, endoplasmic reticulum, Golgi apparatus, lysosome, mitochondria, mitochondrial intermembrane space, nucleus and peroxisome) and the extracellular space (Figure 1A). Furthermore, for each enzyme, the precise sub-cellular location and surface orientation on the membrane site or interface (when a reaction other than a transport is associated with the membrane; *e.g.*, endoplasmic reticulum membrane, cytosolic side) was identified.

### Gap Filling

First, we decided to test whether the model was able to produce biomass by optimising the corresponding reaction accounting for all known biomass precursors required for cell replication. The non-compartmentalised draft model was able to produce biomass without any modifications. In contrast, the compartmentalised draft model was unable to produce biomass. Therefore, we sought to gap fill the draft compartmentalised to identify transporters needed to be added to the model for production of all biomass components. We searched for the minimal number of transporters necessary to add to the model in order to enable the production of biomass. The results of the algorithm were checked manually for two criteria: (i) the result did not suggest a reversible reaction for a known irreversible reaction and (ii) the added reaction transports a metabolite from a compartment where it was produced to a compartment where it was used as reactant.

### Compartmentalised Model

A detailed list of the transporters is provided in Table S1. A small but significant percentage of the reactions are occurring in multiple compartments, in accordance with the experimental data used for the model reconstruction 20%, 3%, and 2% of the reactions were localised to two, three, or a higher number of compartments, respectively; This described 2,286 biochemical reactions, 2,824 compartment-specific reaction (with associated enzymes) and 2,910 compartment-specific, surface-specific reactions (*e.g.*, one cytosolic reaction can occur both on the cytosolic side of the Golgi apparatus and the endoplasmic reticulum membranes). However, even if some reactions occur in more than one compartment, enzymes are reaction- and compartment-specific; No enzyme was described in more than one compartment. Table S2 describes the main pathways, their compartment, and the reaction count.

**Figure 2:**
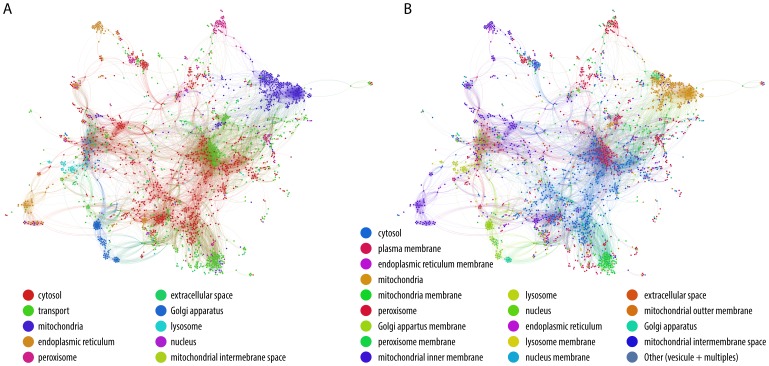
ZebraGEM model (EBI BioModel: MODEL1204120000). (**A**) Compartmentalised network. Each compartment is colour-coded. Nodes represent metabolic reactions, while edges links shared metabolites accounting for the reaction directionality, from product to reactant. (**B**) Surface orientation and interface details. The location of each reaction (excluding transporters) within the compartments and their membranes is detailed.

Figure 1B outlines the model by summarising the reaction and gene content for each subcellular compartmentalisation and metabolite exchanges between compartmentalisation and with the environment. ZebraGEM contains 9 different compartments (Figure 2A). 18 interfaces were used for transport or exchange with the environment, and orientate membrane-associated reaction sides (Figure 2B). There are 47 nutrient uptakes, while oxygen, water and carbon dioxide can freely exchange with the environment. The synthesis of 42 biomass components is summarised in the biomass reaction (Table S3). 1,553 different metabolites have associations with 2,824 reactions, 4,988 genes/enzymes. Only 418 reactions remain without any known enzyme. About 25% of all reactions involve transport between compartments. The resulting network has a density of 0.024, a diameter of 17 and average path length of 3.49. The entire content of *D. rerio* ZebraGEM is freely available Systems Biology Markup Language format [24] as EBI BioModel: MODEL1204120000. To date, it represents the most comprehencise metabolic networks. Compared to ZebrGEM, KEGG database presents only 1,550 individual reactions without compartment data and MetaFishNET 2,523 unique reactions without enzyme association and only a partial compartmentalisation.

**Figure 3:**
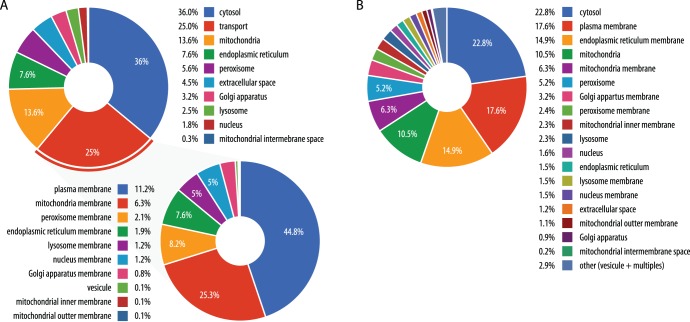
Subcellular localisation of metabolic reactions. (**A**) Distribution of the reaction per compartments or transporters. Transport compartment are detailed by membrane location. (**B**) Details of the reaction distribution (transporters and none transporters) per interfaces (lumen and membrane organelles).

Figures 2A & 3A outlines ZebraGEM model as it reports all reactions distributed in the 9 different compartments and their links (shared metabolites). Figure 2B & 3B details the reactions distributed in the 18 different interfaces (lumen and membrane organelles). We subsequently used the metabolic network model to examine classes of metabolites that are uniquely assigned to a particular compartment, either in their lumen or the membranes (Figure 3). The extensive transport of metabolites between compartments makes such analysis at the network-scale of particular interest.

### Model Validation

Following previous model reconstructions [25]–[27], we further validated the global model reconstruction using a set of simulations of known metabolic functions, such as amino acids, as well as secondary metabolites biosynthesis and degradation (Table S4). All simulations were successful, demonstrating that this model is indeed functional (see Methods).

### Experimental Support

Examination of experimentally characterised pathway activity was used to validate the global metabolism model. For example, the *de novo* biosynthesis of taurine, our model suggests that the only path able to synthesis the taurine is controled by cysteine sulfinic acid decarboxylase. This finding is in accordance with the recently reported critical role of cysteine sulfinic acid decarboxylase in *D. rerio* taurine homeostasis and cardiac development [28]. Likewise, a large portion of the unique metabolites detected in the endoplasmic reticulum were fatty acid-related, including sterols, sphingolipids, and CoAs [29]. In the peroxisome, CoA metabolites were prominent [30]–[33].

### Conclusion

System biology approaches to evolution are attractive as they bring the promise of quantitative analyses of trait evolution and behaviour, integrating phenomena from the molecular to the ecosystem level. As a result, they allow us to describe precise hypotheses as to how traits can be successively used for metabolic engineering.

Several limitations should be noted: (i) As the approach hinges upon different molecular data, its accuracy depends on the quality of the latter. (ii) Several assumptions are used in the reconstruction process, such as the assumption of minimal addition of reactions and directionality relaxation to resolve network gaps, and the assumption of minimal number of metabolite transmembrane transport used in the localisation assignment. These assumptions, although commonly used for similar purposes [34], [35], may in some cases be oversimplified. (iii) The resulting models do not explicitly account for transcriptional and metabolic regulation; data that is still unavailable for most *D. rerio* metabolic functions, and thus cannot accurately predict some of the organism’s functions. It should be noted, however, that further experimental validation should be performed to reaffirm the predictions.

A number of future refinements and extensions will improve the quality of the resulting network models. Such reconstructed models, in addition to those presented here for *D. rerio*, open up future opportunities for metabolic engineering of increased production rates of various nutraceuticals important for human and fish health.

In this work we present the reconstruction of a fully compartmentalised model of *D. reria*, a model fish and present a new feature in metabolic modelling: surface orientation. Despite its evolutionary divergence to farm fish, the genetic make-up of the *D. reria* model fish is the first step toward fish metabolic engineering, making its study highly useful to other fish, as well as to the fish industry, where metabolic engineering is commonly used for quality-trait improvement.

## Methods

### Reconstruction Procedure

An initial component list was assembled as described in the text (see Results and Discussion). Putative gene assignments were collected for KEGG build 39 [19], and verified based on evidence collected from genome annotation databases, namely EntrezGene [21], Ensembl 64 [36] *D. rerio* assembly Zv9, and scientific literature. Extra reactions were collected from MetaFishNET release 1.9 [18]. Substrate and cofactor preferences were identified from the literature and NCI/CADD Chemical Identifier Resolver beta 4 [23] or ChemSpider [37]. Metabolite formulae and charges were calculated based on their ionisation state at pH 7.2, which was assumed to be constant across all compartments. Reaction directionality was determined from thermodynamic data or inferred from empiric data and textbooks. For clarity, we have used only the apparent primary specificity of each enzyme in our network. Compartmentalisation was determined from protein localisation data; sequence targeting signals, and indirect physiological evidence. Transport reactions were entirely reconstructed based on literature reports and biochemistry textbooks because the current annotation of transporters is not sufficiently specific with regard to substrates and mechanisms. Each reaction in our network was manually curated with regard to reaction stoichiometry, subcellular localisation and directionality. By default each particular reaction was defined to be bidirectional, except if explicitly stated otherwise by the literature or the source databases. Based on the controlled nomenclature, the function of a reaction was used to generate descriptive statistics. The entire content of *D. rerio* ZebraGEM, with the references used to describe each reaction, is freely available Systems Biology Markup Language format [24] as EBI BioModel: MODEL1204120000.

Each mapped reaction is defined as a node in our network. Edges between these nodes are defined by shared metabolites between the reactions. The network is directed: for irreversible reactions if the product of one reaction is a reactant in the second, we defined a directed edge. Reversible reactions are treated similarly, except that both directions of the reaction were allowed and handled independently. The networks were visualised with Gephi v0.8 beta [38].

### Exchange Reactions and Biomass Synthesis

Thirteen currency metabolites (H^+^, H_2_O, ATP, ADP, P_i_, PP_i_, Na^+^, coenzyme A, O_2_, NAD^+^, NADH, NADP^+^, and NADPH) were removed from all analyses (but the flux balance analyses) in whichever compartments they occurred [39]. Exchange reactions (Table S2, type ‘import’) allow metabolites to enter and/or leave the metabolic network and were formulated considering the minimum nutritional physiology of the cell. According to published dietary requirement from farm fish [40], the network considers 10 essential amino acids, 2 lipids, 9 carbohydrates, 6 macrominerals, 6 microminerals, 4 fat-soluble vitamins and 10 water-soluble vitamins nutrient uptakes. Likewise, four lipids can be used in fish nutrition; linolenic and linoleic acid, mainly for freshwater fish and eicosapentaenoic and docosahexaenoic acid for seawater fish; since the *D. rerio* in a freshwater fish and the model allows the synthesis of eicosapentaenoic and docosahexaenoic acid, only linolenic and linoleic acid were included. Oxygen, water and carbon dioxide can freely exchange with the environment. Metabolic network biomass is composed of all amino acids (protein), nucleic acids (DNA, RNA) and lipids, including triacylglycerol and cholesterol (Table S2, type ‘biomass’).

### Gap Filling

Gap filling was done by importing the required metabolites from one compartment to another in order to allow all reactions to occur, and subsequently to produce all biomass components. Every metabolite that could not be produced or consumed was manually examined to identify possible reactions describing its degradation, production, or transport. Each added transporter was manually checked for applicability (such as the feasibility of the suggested directionality) and for being reported in at least one other species (fish when available, mammalian otherwise) before been included. We searched for the minimal number of transporters necessary to add to the model in order to enable the production of biomass.

### Flux Balance Analysis

We used the Systems Biology Research Tool v2.0.0 [41] to perform flux balance analysis on the networks [34]. Exchange reactions were added to enable uptake and secretion of extracellular metabolites for the purpose of simulations (Table S5). The optimum flux distribution is here defined as the flux distribution that minimises uptakes for a fixed rate of biomass synthesis. The biomass composition (Table S2) was taken from the published dietary requirement from farm fish [40]. Note that exchange fluxes are negative for influx and positive for efflux by definition.

### Functional Validation

Functional validation was performed by using flux balance analysis. The test simulations validates that the targeted metabolite could be synthetised from a particular source metabolite without violating the other model constraints (*i.e.*, reaction directions, model inputs).

## Supporting Information

Table S1 Detailed list of the transporters.
(CSV)

Table S2 Detailed list of the pathways by compartment and the number of reaction involved.
(CSV)

Table S3 Detailed list the uptakes and biomass reactions.
(CSV)

Table S4 Simulations of known metabolic functions. Complete list of the 160 simulations used to validate ZebraGEM.
(CSV)

Table S5 Exchange reactions added to enable uptake and secretion of extracellular metabolites for the purpose of simulations.
(CSV)

## References

[1] Harris WS, Miller M, Tighe AP, Davidson MH, Schaefer EJ (2008) Omega-3 fatty acids and coronary heart disease risk: clinical and mechanistic perspectives. Atherosclerosis 197: 12–24. 18160071 10.1016/j.atherosclerosis.2007.11.008

[2] Harris K, Fleming J, Kris-Etherton P (2011) Challenges in estimating omega-3 fatty acid content of seafood from US nutrient databases: A salmon case study. Journal of Food Composition and Analysis 24: 1168–1173.

[3] Bendiksen EÅ, Johnsen CA, Olsen HJ, Jobling M (2011) Sustainable aquafeeds: Progress towards reduced reliance upon marine ingredients in diets for farmed Atlantic salmon (*Salmo salar* L.). Aquaculture 314: 132–139.

[4] Aharoni A, Jongsma MA, Bouwmeester HJ (2005) Volatile science? Metabolic engineering of terpenoids in plants. Trends in plant science 10: 594–602. 16290212 10.1016/j.tplants.2005.10.005

[5] Feist AM, Palsson BØ (2008) The growing scope of applications of genome-scale metabolic reconstructions using *Escherichia coli* . Nature biotechnology 26: 659–667. 10.1038/nbt1401 PMC3108568 18536691

[6] Burgard AP, Pharkya P, Maranas CD (2003) Optknock: a bilevel programming framework for identifying gene knockout strategies for microbial strain optimization. Biotechnology and bioengineering 84: 647–657. 14595777 10.1002/bit.10803

[7] Thiele I, Palsson BØ (2010) A protocol for generating a high-quality genome-scale metabolic reconstruction. Nature protocols 5: 93–121. 20057383 10.1038/nprot.2009.203 PMC3125167

[8] Price ND, Reed JL, Palsson BØ (2004) Genome-scale models of microbial cells: evaluating the consequences of constraints. Nature reviews Microbiology 2: 886–897. 15494745 10.1038/nrmicro1023

[9] Becker SA, Feist AM, Mo ML, Hannum G, Palsson BO, et al (2007) Quantitative prediction of cellular metabolism with constraint-based models: the COBRA Toolbox. Nature protocols 2: 727–738. 17406635 10.1038/nprot.2007.99

[10] Henry CS, DeJongh M, Best AA, Frybarger PM, Linsay B, et al (2010) High-throughput generation, optimization and analysis of genome-scale metabolic models. Nature biotechnology 28: 977–982. 10.1038/nbt.1672 20802497

[11] Reed JL, Patel TR, Chen KH, Joyce AR, Applebee MK, et al (2006) Systems approach to refining genome annotation. Proceedings of the National Academy of Sciences of the United States of America 103: 17480–17484. 17088549 10.1073/pnas.0603364103 PMC1859954

[12] Nogales J, Palsson BO, Thiele I (2008) A genome-scale metabolic reconstruction of *Pseudomonas putida* KT2440: iJN746 as a cell factory. BMC systems biology 2: 79. 18793442 10.1186/1752-0509-2-79 PMC2569920

[13] Pal C, Papp B, Lercher MJ, Csermely P, Oliver SG, et al (2006) Chance and necessity in the evolution of minimal metabolic networks. Nature 440: 667–670. 16572170 10.1038/nature04568

[14] Zhang Y, Thiele I, Weekes D, Li Z, Jaroszewski L, et al (2009) Three-dimensional structural view of the central metabolic network of *Thermotoga maritima* . Science 325: 1544–1549. 19762644 10.1126/science.1174671 PMC2833182

[15] Park JH, Lee KH, Kim TY, Lee SY (2007) Metabolic engineering of *Escherichia coli* for the production of L-valine based on transcriptome analysis and *in silico* gene knockout simulation. Proceedings of the National Academy of Sciences of the United States of America 104: 7797–7802. 17463081 10.1073/pnas.0702609104 PMC1857225

[16] Oberhardt MA, Palsson BØ, Papin JA (2009) Applications of genome-scale metabolic reconstructions. Molecular systems biology 5: 320. 19888215 10.1038/msb.2009.77 PMC2795471

[17] Morais S, Pratoomyot J, Taggart JB, Bron JE, Guy DR, et al (2011) Genotype-specific responses in Atlantic salmon (*Salmo salar*) subject to dietary fish oil replacement by vegetable oil: a liver transcriptomic analysis. BMC genomics 12: 255. 21599965 10.1186/1471-2164-12-255 PMC3113789

[18] Li S, Pozhitkov A, Ryan RA, Manning CS, Brown-Peterson N, et al (2010) Constructing a fish metabolic network model. Genome biology 11: R115. 21114829 10.1186/gb-2010-11-11-r115 PMC3156954

[19] Kanehisa M, Goto S, Sato Y, Furumichi M, Tanabe M (2012) KEGG for integration and interpretation of large-scale molecular data sets. Nucleic acids research 40: D109–114. 22080510 10.1093/nar/gkr988 PMC3245020

[20] Webb EC (1992) Enzyme nomenclature 1992: recommendations of the Nomenclature Committee of the International Union of Biochemistry and Molecular Biology on the nomenclature and classification of enzymes: Published for the International Union of Biochemistry and Molecular Biology by Academic Press.

[21] Maglott D, Ostell J, Pruitt KD, Tatusova T (2011) Entrez Gene: gene-centered information at NCBI. Nucleic acids research 39: D52–57. 21115458 10.1093/nar/gkq1237 PMC3013746

[22] Haw RA, Croft D, Yung CK, Ndegwa N, D'Eustachio P, et al (2011) The Reactome BioMart. Database : the journal of biological databases and curation 2011: bar031. 22012987 10.1093/database/bar031 PMC3197281

[23] Sitzmann M, Ihlenfeldt W-D, Nicklaus MC. NCI/CADD Chemical Identifier Resolver: Indexind and Analysis of Available Chemistry Space; 2011 June 5–9 2011; June 5–9 2011, Noordwijkerhout, The Netherlands.

[24] Hucka M, Finney A, Sauro HM, Bolouri H, Doyle JC, et al (2003) The systems biology markup language (SBML): a medium for representation and exchange of biochemical network models. Bioinformatics 19: 524–531. 12611808 10.1093/bioinformatics/btg015

[25] Duarte NC, Becker SA, Jamshidi N, Thiele I, Mo ML, et al (2007) Global reconstruction of the human metabolic network based on genomic and bibliomic data. Proceedings of the National Academy of Sciences of the United States of America 104: 1777–1782. 17267599 10.1073/pnas.0610772104 PMC1794290

[26] Jerby L, Shlomi T, Ruppin E (2010) Computational reconstruction of tissue-specific metabolic models: application to human liver metabolism. Molecular systems biology 6: 401. 20823844 10.1038/msb.2010.56 PMC2964116

[27] Mintz-Oron S, Meir S, Malitsky S, Ruppin E, Aharoni A, et al (2012) Reconstruction of Arabidopsis metabolic network models accounting for subcellular compartmentalization and tissue-specificity. Proceedings of the National Academy of Sciences of the United States of America 109: 339–344. 22184215 10.1073/pnas.1100358109 PMC3252957

[28] Chang YC, Ding ST, Lee YH, Wang YC, Huang MF, et al.. (2012) Taurine homeostasis requires de novo synthesis via cysteine sulfinic acid decarboxylase during zebrafish early embryogenesis. Amino acids. 10.1007/s00726-012-1386-8 22907836

[29] Melville DB, Knapik EW (2011) Traffic jams in fish bones: ER-to-Golgi protein transport during zebrafish development. Cell adhesion & migration 5: 114–118. 21178403 10.4161/cam.5.2.14377 PMC3084976

[30] Tseng YC, Chen RD, Lucassen M, Schmidt MM, Dringen R, et al (2011) Exploring uncoupling proteins and antioxidant mechanisms under acute cold exposure in brains of fish. PloS One 6: e18180. 21464954 10.1371/journal.pone.0018180 PMC3064598

[31] Monroig O, Rotllant J, Cerda-Reverter JM, Dick JR, Figueras A, et al (2010) Expression and role of Elovl4 elongases in biosynthesis of very long-chain fatty acids during zebrafish *Danio rerio* early embryonic development. Biochimica et biophysica acta 1801: 1145–1154. 20601113 10.1016/j.bbalip.2010.06.005

[32] Krysko O, Stevens M, Langenberg T, Fransen M, Espeel M, et al (2010) Peroxisomes in zebrafish: distribution pattern and knockdown studies. Histochemistry and cell biology 134: 39–51. 20556416 10.1007/s00418-010-0712-z

[33] Song Y, Selak MA, Watson CT, Coutts C, Scherer PC, et al (2009) Mechanisms underlying metabolic and neural defects in zebrafish and human multiple acyl-CoA dehydrogenase deficiency (MADD). PloS One 4: e8329. 20020044 10.1371/journal.pone.0008329 PMC2791221

[34] Orth JD, Palsson BØ (2010) Systematizing the generation of missing metabolic knowledge. Biotechnology and bioengineering 107: 403–412. 20589842 10.1002/bit.22844 PMC3119652

[35] Mintz-Oron S, Aharoni A, Ruppin E, Shlomi T (2009) Network-based prediction of metabolic enzymes' subcellular localization. Bioinformatics 25: i247–252. 19477995 10.1093/bioinformatics/btp209 PMC2687963

[36] Flicek P, Amode MR, Barrell D, Beal K, Brent S, et al (2011) Ensembl 2011. Nucleic acids research 39: D800–806. 21045057 10.1093/nar/gkq1064 PMC3013672

[37] Williams AJ, Tkachenko V, Golotvin S, Kidd R, McCann G (2010) ChemSpider - building a foundation for the semantic web by hosting a crowd sourced databasing platform for chemistry. Journal of Cheminformatics 2: O16–O16.

[38] Bastian M, Heymann S, Jacomy M (2009) Gephi: An Open Source Software for Exploring and Manipulating Networks. Third International AAAI Conference on Weblogs and Social Media. Sn Jose, CA: AAAI Publications. pp. 361–362.

[39] Huss M, Holme P (2007) Currency and commodity metabolites: their identification and relation to the modularity of metabolic networks. IET systems biology 1: 280–285. 17907676 10.1049/iet-syb:20060077

[40] Subcommittee on Fish Nutrition, National Research Council (1993) Nutrient Requirements of Fish. Washington, D.C.: The National Academies Press.

[41] Wright J, Wagner A (2008) The Systems Biology Research Tool: evolvable open-source software. BMC systems biology 2: 55. 18588708 10.1186/1752-0509-2-55 PMC2446383

